# Ventral pallidum efferent pathways via mediodorsal thalamus and lateral habenula mediate default mode network regulation

**DOI:** 10.1016/j.isci.2025.113885

**Published:** 2025-10-28

**Authors:** Epistimi-Anna Makedona, Mu-En Kuo, Michael Harvey, Gregor Rainer

**Affiliations:** 1 Section of Medicine, Faculty of Science and Medicine, University of Fribourg, Fribourg, Switzerland

**Keywords:** Neurology, Neuroscience, Behavioral neuroscience, Molecular neuroscience, Cellular neuroscience, Sensory neuroscience, Cognitive neuroscience

## Abstract

Transitions between internal and external focus are fundamental to cognition. These shifts depend on the modulation of the default mode network (DMN), of which the ventral pallidum (VP) is a key subcortical node. Here, we examine which VP efferent pathways mediate these transitions, using projection and cell type specific optogenetic silencing. We found that the inhibition of the VP projection to the lateral habenula (LHb) confers a learning advantage early during task acquisition. Conversely, silencing VP projections to the mediodorsal thalamus (MD) improves performance during the late stage of the same task. The downregulation of the cholinergic VP population improved performance at both early and late stages. Thus, silencing these VP outputs promotes escape from a DMN brain state, facilitating attention to external stimuli. Our results confirm a role for VP in DMN regulation and indicate that MD and LHb VP efferent pathways, in concert with cholinergic neuromodulation, mediate different aspects of DMN regulation.

## Highlights

• VP efferent pathways mediate transitions between DMN and attentional states
• Initiation and maintenance of state transitions rely on different VP target areas
• VP efferents to LHb and MD initiate and maintain state transitions, respectively
• VP cholinergic neurons impact both the initiation and maintenance of state transitions

## Graphical abstract

**Figure:**
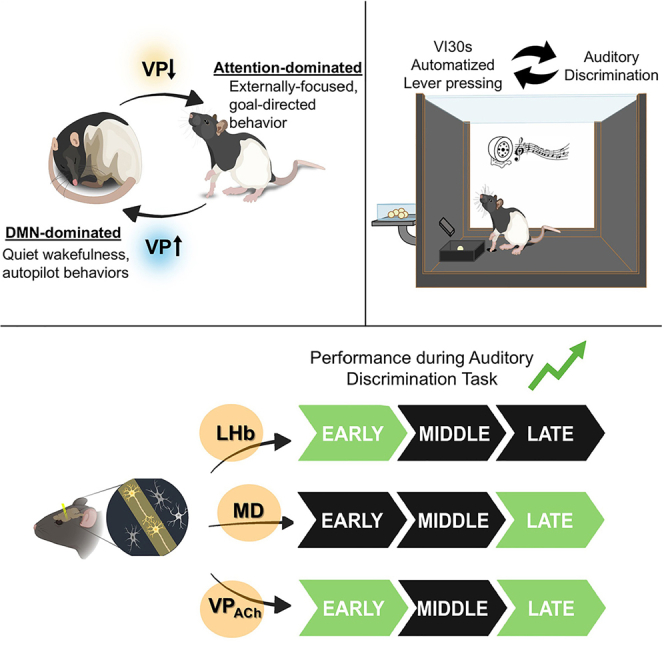

## Introduction

The default mode network (DMN) is a large-scale brain circuit that is active during quiet wakefulness and deactivated during the performance of goal-directed cognitive tasks involving attention directed to the external world.^1^^,^^2^ During quiet wakeful states, the DMN is thought to support various functions including introspection, mnemonic processing, and autobiographical mental activity. In addition, human imaging work has revealed that the DMN is also activated during highly repetitive, “autopilot” behaviors, which do not involve attention to external stimuli and can thus be considered as largely endogenously driven.^3^^,^^4^^,^^5^ While introspection is difficult to study in preclinical animal models, transitions from “autopilot” to attentionally demanding tasks are feasible in animals and can provide useful insights into maintenance and transitions into DMN brain states. Indeed, the DMN is highly conserved among mammalian species, and, in addition to humans, has been described in rodents, tree shrews, and non-human primates.^6^^,^^7^^,^^8^

It has become apparent that the DMN encompasses not only cortical areas such as the medial prefrontal and cingulate cortices and surrounding areas of the temporal and parietal lobes, but also a number of subcortical structures.^9^^,^^10^^,^^11^^,^^12^ Convergent evidence suggests that notable subcortical DMN regions include the nuclei of the basal forebrain in addition to other structures such as the thalamus. On the one hand, this evidence comes from human fMRI studies, which have identified both the basal forebrain and mediodorsal thalamus as critical subcortical DMN nodes based on functional and structural connectivity^10^^,^^12^ and dynamic causal modeling.^13^ This work is complemented by preclinical studies in animals, which provide corroborating evidence for the results in humans and in addition permit delineating the role of specific cell-types in DMN regulation.^14^^,^^15^^,^^16^ Thus, the parvalbumin (PV) positive GABAergic neurons of the basal forebrain (BF), magnocellular preoptic (MCPO) nucleus contribute to DMN activation, in that the optogenetic activation of these neurons activates cortical DMN nodes and elicits DMN-associated behaviors.^17^ Consistent with this, the excitation of the BF PV neural population impairs auditory cortical activations during auditory stimulation, potentially compromising auditory-based behavior.^18^ Similarly, somatostatin (SST) positive interneurons in the ventral pallidum (VP) regions of the BF play a key role in regulating cortical DMN gamma oscillations.^19^^,^^20^ A key role of the VP nucleus is also supported by additional studies, showing remarkable gamma oscillations in VP during quiet wakefulness that are reduced during exploration or cognitive task performance.^11^ In another study, the optogenetic activation of BF VP neural activity impaired transitions from automatized lever pressing to an auditory discrimination task, while the deactivation of the same neural population aided such transitions.^21^ Interestingly, this latter study demonstrated a bidirectional modulation of task switching and a robust modulation of lever pressing rate, raising the question whether these two effects might stem from different cell types or projection pathways of the BF VP nucleus. Our aim here is to identify the pathways by which VP activation or inhibition modulates cortical DMN activity.

The output efferent pathways of the VP have been extensively studied,^22^ and include robust direct projections to medial prefrontal cortex (mPFC) as well as the mediodorsal thalamus, lateral habenula, basolateral amygdala, and a number of brainstem neuromodulatory centers. We chose to focus on three of these major VP efferent pathways, namely the cholinergic corticopetal projection, and the projections to the mediodorsal thalamus (MD) and lateral habenula (LHb). The corticopetal VP projections might serve to directly activate target regions in mPFC such as cingulate or prelimbic cortical areas that are part of the DMN. We have previously modulated VP projections using an hSynapsin (hSyn) construct, which targets multiple VP cell types.^21^ Here, we used a ChAT-Cre rat model to specifically activate VP cholinergic neurons, of which a large majority indeed projects to the cortex while a minority projects to subcortical regions.^23^^,^^24^^,^^25^ The MD thalamus is another major target of the VP, receiving VP inputs that are mostly GABAergic,^26^^,^^27^ although there have been suggestions of a cholinergic contribution; these have not been unequivocally validated.^22^^,^^28^ The MD thalamus is the main thalamic counterpart of the prefrontal cortex, and plays an important role in sustaining cortical network activations in different brain states involving the prefrontal cortex, including those linked to the DMN.^10^^,^^29^ The LHb receives a robust projection from the VP^23^^,^^30^^,^^31^ that is largely glutamatergic and GABAergic but also includes a less pronounced cholinergic component.^23^ While our final target, the LHb, does not project directly to cortical DMN areas, it does exert substantial influence on ascending neuromodulatory projections, in particular serotonergic and dopaminergic systems, which can in turn profoundly modulate cortical state and potentially trigger DMN activation.^32^^,^^33^^,^^34^ Indeed, it has been demonstrated that the optogenetic inhibition of the LHb in rats triggers a reduction in the connectivity within DMN structures,^35^ suggesting that the LHb may serve to maintain a DMN brain state. A role of the LHb in DMN activation is also generally consistent with the general view that the LHb is crucial for generating dynamic adaptive behaviors in changing contexts and environmental conditions.^32^^,^^36^

Taken together, our goal here is to examine to what extent these three pathways contribute to the influence that the VP nucleus exerts on triggering and maintaining a DMN brain state. We first address this question using electrophysiological recordings in the anesthetized animal to assess the impact of the optogenetic modulation of efferent VP pathways on neural circuit activity in target regions. Then, we examine to what extent manipulating these pathways impacts operant behavior during transitions between internally focused, automatized lever pressing and an auditory discrimination task requiring externally focused attention. Finally, in awake animals performing the same task, we explored the local field potential (LFP) activity in the putative DMN structures in VP, MD, and ACC, as well as a control area, the auditory cortex (AC).

## Results

### Functional validation of ventral pallidum AAV-hSynapsin-ChR2 virus injections

**Figure 1:**
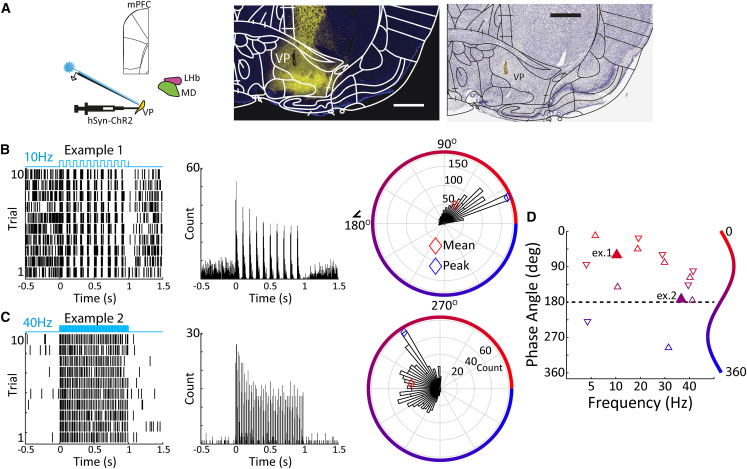
Functional validation of VP AAV-hSyn-ChR2 virus injections (A) (left) Schematic of the virus injection and stimulation/recording sites (VP ventral pallidum, LHb lateral habenula, MD mediodorsal nucleus, mPFC medial prefrontal cortex). (center, right) example immunofluorescence and Nissl-stained sections illustrating viral expression and probe placement in the VP. (B) Action potential raster plot, peri-stimulus-time-histogram, and spiking phase angle histogram in relation to optogenetic stimulation frequency for an example stimulation site at 10 Hz light stimulation frequency. Red and blue symbols represent the mean and peak preferred phase angle, respectively. (C) same as (B) for an example unit at 40 Hz light stimulation. (D) Population analysis across VP stimulation sites of mean preferred phase angles, illustrating unit responses entrained to the onset of light stimulation (upward pointing and downward pointing triangles denote units with overall excitation and inhibition relative to pre-stimulation baseline, respectively).

Here, our overall strategy is to perform optogenetic neural circuit manipulations to functionally validate VP efferent pathways to MD and LHb, and determine whether activating these pathways modulates activity in DMN cortical areas. Following neural circuit validation, the role of these pathways in regulating behavior is assessed. In a first step, we assessed how the optogenetic activation of VP neurons impacts activity in the LHb by injecting AAV-hSyn-ChR2-mCherry into the VP and recording spiking activity in VP and LHb during pulsatile (5, 10, 20, 30, and 40 Hz) blue (473 nm) laser stimulation of the VP. We first established that VP light stimulation modulated local neural activity, and verified optrode placement as well as viral expression in the VP using Nissl stained sections and epifluorescence microscopy, respectively (Figure 1A). Our strategy was to first lower an optrode into the VP until we found unit activity modulated by laser stimulation. Once such responses were encountered, the optrode was generally left in place while we recorded responses in LHb. Within VP, we obtained data from 8 stimulation sites in 4 rats, where neurons were significantly modulated by laser stimulation, with 5 showing net excitation relative to baseline activity and 3 showing net inhibition (paired t-tests or Wilcoxon signed-rank test, depending on normality, see methods, *p* < 0.05). Raster plots and peri-stimulus time histograms (PSTHs) for two VP neurons that were excited by laser stimulation are shown in Figures 1B and 1C. To confirm that VP units were excited by laser onset, we calculated phase histograms of spiking activity relative to the stimulation frequency. Both example units were indeed phase-locked (Rayleigh test, *p* < 0.05) to the laser onset with phase angles consistent with activity during laser stimulation. Across all recorded VP stimulation sites, we observed 15 cases of significant unit phase locking across stimulation frequencies, with some units showing phase locking at multiple frequencies (Figure 1D). As was the case for the two examples, units tended to fire preferentially during light-on phase angles, i.e., less than 180° (13/15 cases). Taken together, this indicates that rat VP neurons are readily transfected by AAV virus containing the hSyn promoter, can be activated at short latencies across a range of stimulation frequencies, and are preferentially phase locked mainly to the light-on periods of pulsatile laser stimulation. In addition, 40 Hz optogenetic VP stimulation robustly drives local circuit activations in the frequency range previously linked to DMN behavior.^11^

### Ventral pallidum activation drives the lateral habenula

**Figure 2:**
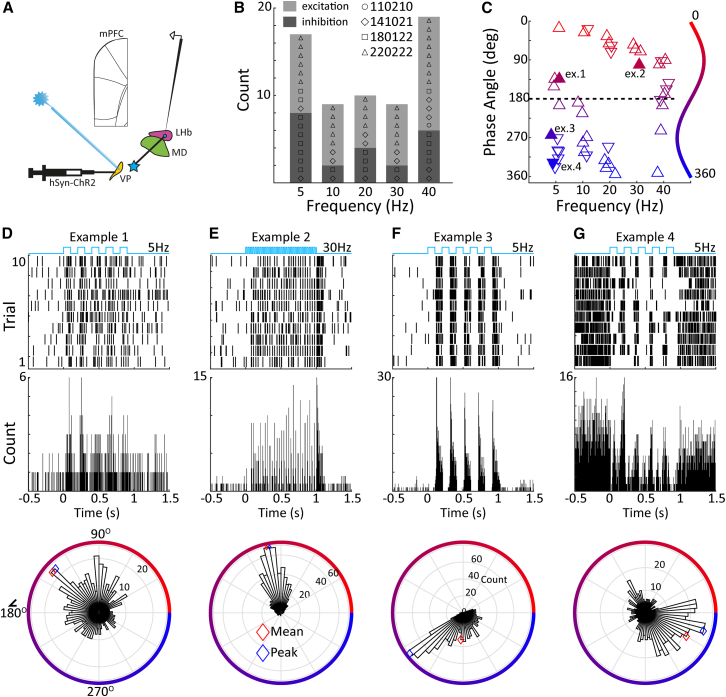
Functional validation of the VP to LHb pathway (A) Schematic of the virus injection and stimulation/recording sites. (B) Histogram of overall excitation and inhibition encountered in LHb following VP light stimulation at different frequencies. (C) Spiking phase angle histogram for LHb units, illustrating response entrainment to light onset at angles below 180° as well as frequent responses to light offset at angles greater than 180°, consistent with rebound excitation following the transient inhibition of LHb during VP stimulation (upward and downward pointing triangles denote units with overall excitation and inhibition relative to pre-stimulation baseline, respectively). (D and E) Action potential raster plot, peri-stimulus-time-histogram, and spiking phase angle histogram in relation to optogenetic stimulation frequency for an example stimulation site at the indicated stimulation frequency for two units (ex., 1,2 in panel C) showing overall excitation during light stimulation compared to baseline. Red and blue symbols represent the mean and mode preferred phase angle, respectively. (F and G) Same as (D and E) but for two units showing inhibition during light stimulation (ex.3,4 in panel C).

With functionally validated VP optrode placement, we recorded from 58 well isolated single units in the LHb (Figure 2A). Of these, 30 significantly modulated their firing rates (paired t-tests or Wilcoxon signed-rank test, *p* < 0.05) in response to VP optogenetic stimulation, with 63% (*n* = 19) exhibiting net excitation and 37% (*n* = 11) exhibiting net inhibition. The bidirectional modulation of neural activity was also observed when examining all cases of significant modulation across stimulation frequencies (Figure 2B). We observed that 24 of these units were significantly phase locked to the stimulation at at least one of the frequency conditions, i.e., 5, 10, 20, 30, or 40 Hz, (Rayleigh test, *p* < 0.05). Examining phase-angle preference (Figure 2C), we found 23 cases with a preference for light-on phase angles, similar to our observations in VP and consistent with a glutamatergic influence of VP on LHb.^31^ Neural activity and phase preference of two example neurons are shown in Figures 2D and 2E. In contrast to VP, there was a considerable neuronal population in LHb showing preference for light-off phase angles (*n* = 20). Two example LHb neurons are shown which exhibit overall net-excitation (Figure 2F) and inhibition (Figure 2G), but during the activation of VP, are consistently activated during light-off periods. Such rebound bursting has been previously demonstrated in LHb following transient inhibition,^37^ consistent with GABAergic inputs from VP. In summary, VP activation drives LHb via direct as well as rebound excitation. We note that with different populations of LHb neurons firing at both phases of pulsatile stimulation, downstream LHb targets of convergent input from both populations would receive constant excitatory drive during periods of oscillatory VP activation.

### Ventral pallidum optogenetic stimulation entrains the lateral habenula at 40 Hz

**Figure 3:**
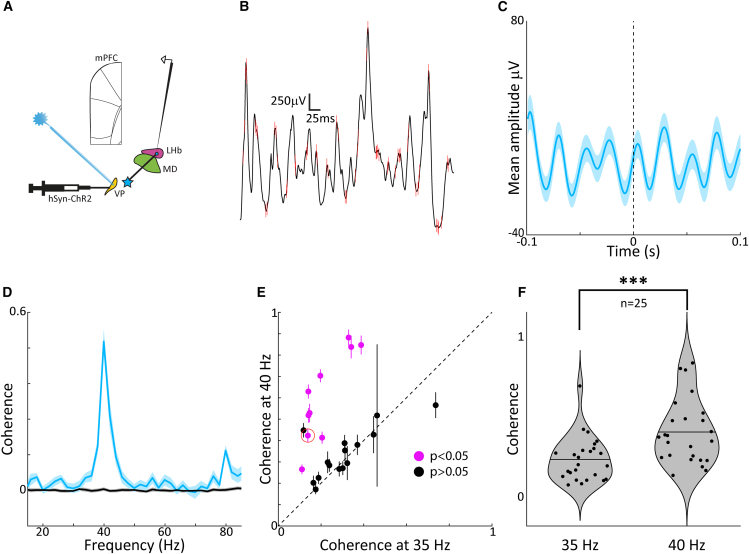
Functional connectivity of VP to LHb pathway (A) Schematic of the virus injection and stimulation/recording sites. (B ) Example VP spike times overlaid on LHb LFP and (C) The mean VP spike triggered average of the LHb LFP (±SEM, shaded) suggests a systematic impact of VP on LHb neural circuit activity. (D) The spike-LFP coherence for an example session shows specificity to the light stimulation frequency (40 Hz) over other non-stimulation frequencies. (E and F) Across the population of VP-LHb recording sites, 10/25 sites showed significantly elevated coherence at 40 Hz compared to a control frequency of 35 Hz (*p* < 0.05), and (F) overall there was a significant effect across the entire population of recording sites (∗ symbol denotes significance, paired t-test: p≪0.05). Error bars represent the standard error of the mean (SEM).

Given the prevalence of gamma oscillations in the VP during DMN brain states,^8^^,^^11^^,^^21^ we further characterized the influence of VP on LHb during 40 Hz optogenetic stimulation by examining the temporal relationship between action potential timing in VP and local field potential (LFP) oscillations in LHb (Figure 3A). The examination of an example segment of data (Figure 3B) suggests a tendency for VP action potentials to occur near the depth positive peak of the LHb LFP. Such a systematic relationship provides evidence for a functional interaction between VP and LHb, which is further supported by the observation that the VP spike triggered-averaged LFP in LHb exhibits robust oscillations at the stimulation frequency, 40 Hz (Figure 3C). Computing the spike-field coherence (see methods) for this same recording session, we observe a notable peak at 40 Hz, as well as a first harmonic at 80 Hz, further supporting the notion that the optogenetic activation of VP robustly activates LHb neural circuits. A population analysis comparing spike-field coherence at the stimulation frequency (40 Hz) to an adjacent control frequency (35 Hz) highlights the robustness of the influence of VP activation on LHb circuit activity (Figures 3E and 3F), with 11/25 recording sites showing significant elevation in coherence (paired t-test or Wilcoxon signed-rank test, *p* < 0.05) and also an overall significant effect across the population of 25 sites (Wilcoxon signed-rank test: p≪0.01). These provide functional evidence for an interaction between VP and LHb at 40 Hz.

### Activation of lateral habenula/mediodorsal thalamus and medial prefrontal cortex targets following ventral pallidum terminal stimulation

**Figure 4:**
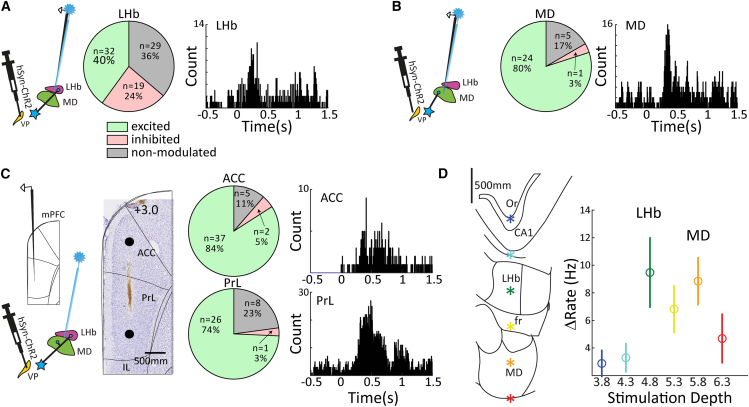
Activation of LHb/MD and mPFC targets following VP axonal terminal stimulation (A) Light stimulation of VP axons in LHb elicited local circuit activity. The pie chart shows significantly excited and inhibited units (*p* < 0.05 compared to baseline activity), and an example PSTH of a modulated LHb unit. (B) same as (A) but for the MD thalamus. (C) Neural activation observed in ACC and PrL regions of mPFC following the light stimulation of VP axon terminals in LHb/MD, highlighting largely excitatory influence of this pathway on the studied DMN cortical areas. Pie charts show significantly excited and inhibited units (*p* < 0.05 compared to baseline activity), and an example PSTH is shown for a unit in each cortical area. (D) Enhancement of mPFC activity occurred selectively for light stimulation in LHb and MD, but not for stimulation in the hippocampus (one-way ANOVA on firing rate increase over baseline as a function of stimulation depth, *p* < 0.05). Error bars represent the standard error of the mean.

In order to understand the impact of VP activation on DMN cortical areas in the mPFC via the LHb and MD pathways, we injected AAV-hSyn-ChR2-mCherry into VP and allowed at least 60 days for viral expression. We then stimulated the axons of VP projections in LHb and MD with blue light, while recording neural activity in LHb/MD and mPFC regions, anterior cingulate (ACC) and prelimbic (PrL) cortex (Figure 4A). Recordings in ACC were made using laminar electrodes with 16 channels spaced at 100 μm, while recordings from LHb and MD were made with a similar laminar probe but equipped with 3 LED light guides spanning the depth of the probe. Light stimulation was performed at five frequencies (5, 10, 20, 30, and 40 Hz) with a 50% duty cycle. We found that light stimulation in the LHb and MD modulated local units, *N* = 51 and *N* = 25, respectively (paired t-test or Wilcoxon signed rank test of activity during stimulation compared to baseline, *p* < 0.05, Figures 4A and 4B). Stimulating the axonal terminals from VP in LHb triggered mixed excitation and inhibition in LHb, similar to the stimulation of VP somata (Figure 2), although the responses following axonal terminal stimulation exhibited far less phase entrainment (3/54 units or 5%, Rayleigh test, *p* < 0.05) to light stimulation. In MD, VP axonal terminal stimulation triggered mostly excitatory responses when considering the entire 1s stimulation period. However, these excitatory responses were preceded by an initial low-latency inhibition in a significant fraction of neurons, such that of the 24 overall activated MD units, 18 were transiently inhibited during the first 100 ms of stimulation while only 3 showed excitation during this period (χ^2^-test, *p* < 0.001). Activating the VP to MD pathway thus has an overall excitatory impact on MD activity, and by extension, cortical MD target regions belonging to the DMN, despite the fact that this projection is GABAergic and produces initially inhibitory responses. This response pattern has indeed been previously observed following the electrical stimulation of VP,^26^^,^^27^ and is supported by intracellular recordings showing IPSPs followed by EPSPs in MD following VP stimulation, also confirming a largely GABAergic nature of this pathway.^38^ In addition to modulating activity in MD and LHb, the activation of VP axon terminals in these areas also resulted in the robust modulation of cortical DMN areas, prelimbic (PrL), and anterior cingulate (ACC) cortex. As illustrated in Figure 4C, the activation of the subcortical VP pathways triggered primarily excitatory responses in DMN cortex (paired t-tests or Wilcoxon signed-rank test, evoked vs. baseline activity, *p* < 0.05). We next examined the impact of stimulation localization on the strength of the mPFC response by dividing all stimulation depths into 500 μm bins and averaging the mPFC cortical response for each site. Figure 4D shows the location of our bin centers and the resultant responses, highlighting that mPFC activations selectively occurred for stimulation within LHb/MD but not adjacent areas along the stimulation track (one way ANOVA for stimulation depth, *p* < 0.05). Taken together, these experiments show that the selective stimulation of the VP to LHb and VP to MD pathways triggers activation both locally in these subcortical structures as well as in mPFC regions, consistent with the hypothesis that these pathways contribute to the maintenance of the DMN.

### Pathway specific inhibition of ventral pallidum afferents has dissociable effects on behavior

**Figure 5:**
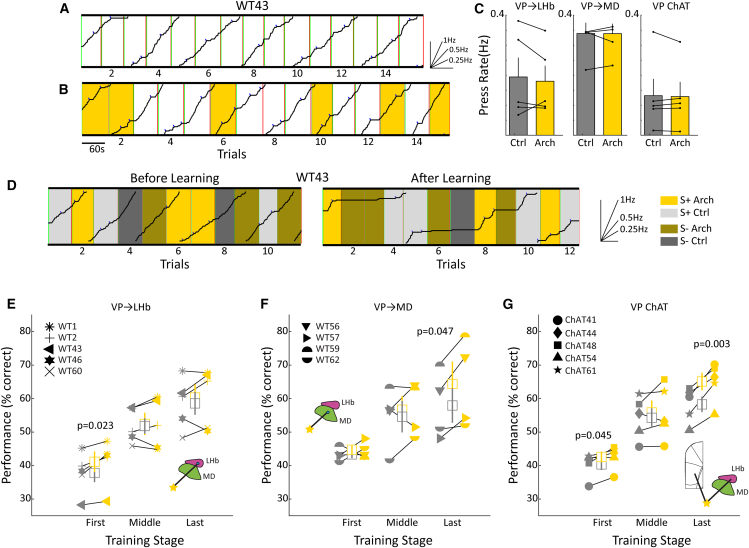
Pathway specific inhibition of VP afferents has dissociable effects on behavior (A) A cumulative record of lever pressing for an animal working for food rewards on a VI-30 schedule of reinforcement. Each upward deflection in the record indicates a lever press, and blue hash marks indicate rewards. The slope of the record, thus, is indicative of response rate, inset. The records reset to zero after 50 lever presses, and green and red vertical lines indicate trial start and stop times, respectively. Plotted is typical behavior on this schedule of reinforcement, that is, a slow and stable response rate. (B) An example cumulative record for an animal receiving optogenetic inhibition of VP ChAT neurons during VI-30 lever pressing, demonstrating a lack of effect of the optogenetic intervention on lever press rate. (C) The group results of optogenetic inhibition on lever press rate for the three projection systems studied, showing a lack of effect on response rates in each case. Error bars represent the standard error of the mean. (D) Example cumulative records of lever pressing for the same animal during the auditory discrimination task. Left, the cumulative record before task acquisition showing that the rat keeps pressing at the same rate during rewarded (S+) and unrewarded (S-) trials. Right, the cumulative record after task acquisition shows the reduced lever press rate during unrewarded trials. (E) The performance of individual rats during the three different training stages (first, middle, and last) of an auditory discrimination task for control trials (gray) and trials with optogenetic silencing of the VP to LHb projection, yellow; *p* value shown, repeated measures ANOVA. (F) Same as E, but for the optogenetic silencing of the VP to MD projection. (G) Similarly, but for the optogenetic inhibition of the cholinergic population of VP. For panels (E–G), error bars represent the standard error of the mean.

Having functionally validated that the VP to the LHb pathway engages cortical DMN areas, we proceeded to study the effect of the optogenetic deactivation of this pathway on automatized lever pressing behavior and task switching. We therefore injected AAV-hSyn-Arch-eYFP into VP and illuminated the VP axonal terminals in LHb using a wireless fully implantable device (see methods and Sup.Figure S1) at 40 Hz. In addition, we also studied the VP to MD pathway as well as cholinergic VP efferent projections using virus injections AAV-retro/2-hSyn-Arch and AAV-hef1a-dlox-Arch, respectively. After suitable training (see methods), rats lever pressed on a variable interval, VI30s, reinforcement schedule, which served as a model of an automatized, “autopilot” behavior involving DMN activation.^4^ On VI schedules of reinforcement, rewards are delivered for the first lever press following a randomized time interval, in this case 30 s +/− 5 s. Under these conditions, rats exhibit a steady response rate that is endogenously generated and resistant to reward devaluation or contingency changes,^39^ see example trace Figure 5A. We next inhibited VP axonal terminals in LHb (Sup.Figure 1A) during VI30s lever pressing. As shown in the example trace (Figure 5B) light stimulation appeared to have no effect on lever pressing. This result was confirmed by a population analysis (Figure 5C left) across *n* = 5 rats (paired samples t-test, *p* > 0.1). Similarly, lever press rate during VI30s was also unaffected by the inhibition of VP to MD projections or the inhibition of cholinergic neuron somata in the VP (Figure 5C center, right). Taken together, the robust suppression of VI30s lever press rates observed during the general inhibition of VP somata using AAV-hSyn-Arch^21^ was not recapitulated by any of these manipulations, suggesting that this effect originates from a pathway or combination of pathways not investigated here.

After the completion of VI30s data acquisition, the task was switched to auditory discrimination (AD), where an auditory stimulus (classical music piece) was played during each block, signifying whether rewards continued to be available (S+ block) for lever pressing on a VI30s schedule or no reward was available (S- blocks). During each session, rats encountered blocks with optogenetic manipulation as well as control blocks, allowing us to estimate the impact of the different neural pathways on transitioning from an automatized task under endogenous control to an attentional task where behavioral output depended on sensory auditory stimuli.

We first divided the learning stages into first, middle and last, defined as the first, middle and last seven days of training. We then performed two-way ANOVAs for the main effects of optogenetic intervention or training day on the animals' performance measured as percent correct. We found that the optogenetic inhibition of any of the three pathways resulted in a significant and positive impact on the acquisition of the discrimination task, albeit at different times during learning. The optogenetic silencing of the VP→LHb pathway during the first seven days of training significantly improved the acquisition of the AD, with no effect of training day nor a significant interaction between optogenetic inhibition and training day, [*F*_(1,6)_ = 12.85, *p* < 0.05; *F*_(1,6)_ = 3.21, *p* > 0.05]. That is, it facilitated the escape from automatized behavior due to the suppression of the DMN (Figure 5E).

The optogenetic silencing of the VP→MD pathway had an effect during the last 7 days of learning, again with no effect of day nor a significant interaction between training day and silencing, [*F*_(1,6)_ = 10.65, *p* < 0.05; *F*_(1,6)_ = 0.77, *p* > 0.05]. During this training stage, the animals had already partially acquired the AD (Figure 5F). Finally, the optogenetic inhibition of cholinergic neurons in the VP (Sup.Figure 1B) produced significant effects both early, [*F*_(1,6)_ = 8.34, *p* < 0.05; *F*_(1,6)_ = 2.54, *p* < 0.05] and late, [*F*_(1,6)_ = 38.83, *p* < 0.005; *F*_(1,6)_ = 3.80, *p* < 0.01] with a main effect also of training day but no interaction at both of these learning stages (Figure 5G). Interestingly, the activation of the VP→MD pathway using ChR2 had no effect on the animals' performance at any training stage, nor on the rate of lever pressing (data not shown). These data suggest that VP projections to LHb and MD contribute to DMN triggering and maintenance, respectively, as the disruption of these pathways facilitates attention to external stimuli. Cholinergic VP efferent pathways, which project to mPFC DMN areas as well as to LHb, produce similar effects, and indeed cholinergic effects occur in both the first and last phases of learning.

### Automatized lever pressing is associated with default mode network activation

**Figure 6:**
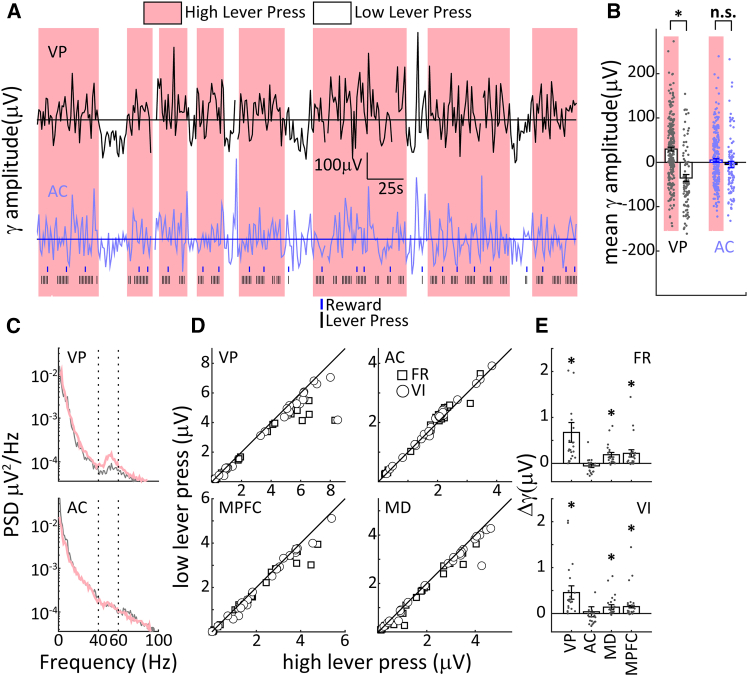
Gamma band activity is elevated in VP, MD, and mPFC but not in AC during high lever pressing (A) Example LFP segments of gamma band (40-60 Hz) in VP (top) and AC (bottom) during high lever pressing periods (pink) and low lever pressing periods (white). (B) Comparison of overall gamma band amplitude for the session in (A) during high lever pressing and low lever pressing in VP (left) and AC (right), ∗ symbol denotes significance (Wilcoxon rank-sum test: *p* < 0.01). Error bars represent the standard error of the mean. (C) Example recording session illustrating a peak in the gamma band (40-60 Hz) power in VP, but not AC. The peak was more prominent for high lever pressing (red) compared to low lever pressing (gray). (D) Population analysis shows that gamma band activity was significantly higher in VP, mPFC, and MD during high lever pressing epochs compared to low lever pressing epochs, but this was not the case for AC (FR, squares; VI, circles, Wilcoxon signed-rank tests: *p* < 0.001). (E) Bar plots showing gamma band activity during high lever press and low lever press for VP, MD, and mPFC, and AC during FR sessions (top) and VI sessions (bottom). ∗ symbols denote significance (sign-tests with Bonferroni correction: *p* < 0.01). Error bars represent the standard error of the mean.

**Figure 7:**
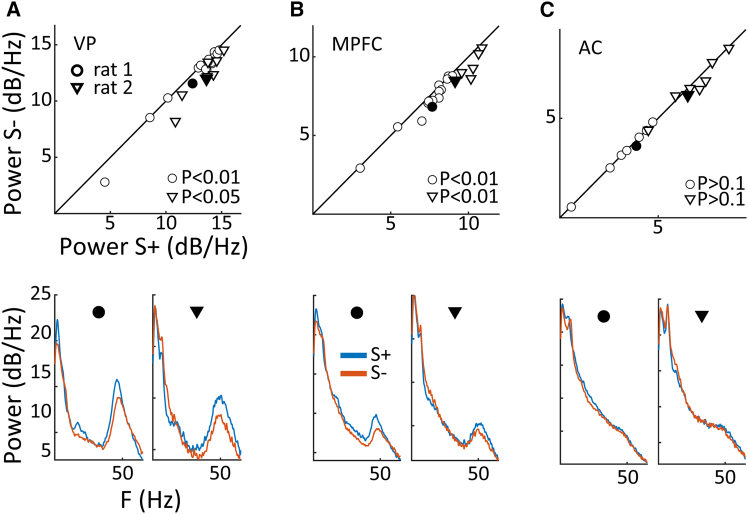
Gamma band power during S- and S+ conditions in 3 brain areas (A) Top, gamma band power in VP during S+ (rewarded conditions) vs. S- (non-rewarded conditions) for 2 rats. *p*-values denote the significance of the Wilcoxon signed-rank test. Bottom, power spectral density (PSD) calculated from LFPs recorded in the VP for S+ (blue) and S- (orange) conditions in the same two animals as above. The sessions correspond to the filled symbols on the top panel. (B and C) Same as (A) but for recordings from mPFC and AC, respectively.

Previous work suggests that enhanced gamma oscillations in the basal forebrain and mPFC are associated with DMN activation.^11^ We therefore wanted to examine whether this held true in the context of task switching and trained a separate group of rats (*n* = 3) for electrophysiological recordings during fixed ratio, FR, VI, and AD tasks (see methods). We recorded from three putative DMN sites, VP, mPFC, and MD, as well as the auditory cortex (AC) as a control site outside the DMN. An example segment of gamma-band (40-60 Hz) power extracted from the VP LFP is shown in Figure 6A for a rat on an FR-10 schedule of reinforcement, with identified periods of high and low lever press activity (see methods). In VP, pauses in lever pressing are accompanied by transient reductions in gamma power, while this effect does not appear to occur in AC (Figure 6A). This trend is confirmed for this dataset in Figure 6B, and these effects were indeed significant across the population of recordings in VP, but not in AC. Example power spectral density (PSD), for VP and AC from a single recording reveals a significant enhancement of gamma band activity in VP but not AC (Figure 6C), and indeed gamma band power during periods of low lever pressing was significantly enhanced in VP, mPFC, and also MD but not AC in the population (Wilcoxon rank-sum tests; *p* < 0.01, Figures 6D and 6E). These findings support the notion of lever pressing as an automatized, DMN behavior. We also examined gamma band activity in the AD task in a subgroup of rats (*n* = 2, see methods), focusing on aggregate activity during S+, and S- blocks. On S+ blocks, rats continue to receive rewards under the same VI30s reinforcement schedule as prior to task switching and thus persevere in automatized lever pressing. On S- trials, however, no rewards are available, and automatized lever pressing behavior is reduced, consistent with a reduction in DMN activity. This reduction was reflected in reduced gamma band power in both VP and mPFC during S- compared to S+ trials (Figures 7A and 7B), whereas no such difference was apparent in AC (Figure 7C). The association of behavioral and electrophysiological results thus supports the notion that automatized lever pressing is associated with DMN activation and that behavioral contexts that require attention to external sensory information are accompanied by reduced DMN activity.

## Discussion

We have implicated a number of efferent pathways originating in the VP in default mode network (DMN) regulation by using specific optogenetic manipulations during task-switching from automatized lever-pressing to a goal-directed auditory discrimination task in the rat. Rather than through a unitary mechanism, our data show that the VP recruits distinct pathways to support specific aspects of DMN regulation. Consistent with earlier work,^21^ we show here that the optogenetic deactivation of these VP efferent pathways improves task-switching, whereas the overwhelming majority of available studies have highlighted deficits in task-switching following lesions or deactivations of these pathways, but see.^40^ As outlined below, we attribute this to the specific transition from an overtrained and automatized internally guided to an externally guided behavior that we studied here. Note that we have focused on two important prefrontal cortical DMN nodes, PrL and ACC, in the present study. Further work is necessary to establish how these activations impact other cortical DMN areas.

Our data show that the functional consequence of VP to MD pathway stimulation is an overall activation composed of an initial inhibition followed by a prominent excitation, as previously observed and consistent with the largely GABAergic nature of this pathway.^26^^,^^27^^,^^38^ Silencing this pathway may likely reduce excitatory drive to the DMN via the MD. Indeed, our behavioral results are compatible with this idea, as with an inactivated VP to MD pathway, rats are less likely to revert to automatized lever pressing, improving performance on the auditory discrimination task. Interestingly, the performance enhancement occurred only during the late stage of learning, where the animals had already partially acquired the task, consistent with a reduction of the ongoing influence of the DMN on behavioral state. This result is largely compatible with the extensive literature implicating the MD's various functions, including behavioral choice selection and cognitive flexibility.^29^^,^^41^^,^^42^ For example, lesions of the MD impair task switching between visual stimulus and egocentric response based strategies in a goal-directed maze navigation task.^43^ The optogenetic silencing of MD also compromises task-switching between visual and auditory guided operant behavior.^44^ Similarly, MD lesions also interfere with goal-directed behavior such that animals are less sensitive to reward devaluation.^45^ Overall, MD lesions compromise cognitive flexibility in multiple behavioral paradigms. In contrast, we found that the manipulation of the VP to MD pathway enhanced behavioral performance for task switching from an automatized to a goal-directed behavior. We suggest, therefore, that the inputs from the VP to MD specifically target neural circuits belonging to the DMN, and deactivating these circuits confers a behavioral advantage in tasks requiring attention to the external environment, as previously demonstrated for the deactivation of neural activity within VP.^21^ Previous work has also shown that gamma oscillations occurring in rodent VP, MCPO, and ACC are a feature of DMN network activation,^17^^,^^21^ and occur during behavioral states linked to the DMN, including quiet wakefulness, self-grooming and automatized operant behaviors. Here, we extend these findings to the MD thalamus by linking MD gamma power to operant lever pressing and revealing reduced levels of gamma during auditory discrimination compared to automatized lever pressing. Related electrophysiological findings have been reported during the acquisition of lever pressing in the rat.^46^ Our findings further support the notion that MD is an important subcortical node of the DMN in the rodent, consistent with previous results in tree shrews and humans.^8^^,^^10^^,^^13^

For the VP to LHb projection, we found a mixture of excitation and inhibition evoked in the LHb using the hSyn-ChR2 construct in VP, consistent with the documented transfection of GABAergic as well as glutamatergic neurons, while being only weakly expressed in cholinergic neurons.^21^ This finding is compatible with previous *in vitro* results, showing short-latency EPSPs or IPSPs in LHb evoked by ChR2 stimulation of VP glutamatergic and GABAergic neurons, respectively.^31^^,^^47^^,^^48^^,^^49^ We frequently observed inhibition followed by rebound bursts of activity in LHb following activation of VP projections. Indeed, such rebound bursts have been documented in LHb in response to local inhibition,^37^ and have been shown to depend on intrinsic cellular properties as well as synaptic input. Yang and colleagues have linked these rebound bursts with depressive disorder, compatible with our hypothesis that the VP to LHb pathway contributes to DMN regulation. Note that depressive disorder has been previously linked to an overactivation of the DMN, where this brain state becomes overly dominant and largely suppresses goal-directed and exploratory activities.^35^^,^^50^ Our finding of a robust functional interaction between VP and LHb at 40 Hz further supports the notion that the LHb plays a role in DMN regulation, as gamma frequency oscillations are a hallmark of DMN activity in cortical and subcortical structures.^11^^,^^17^^,^^19^^,^^51^^,^^52^

In terms of behavior, the deactivation of the VP to LHb pathway improves task-switching out of default mode and into an attentional, goal-directed behavioral state. In contrast to the MD mediated effects, the LHb pathway produced effects early during the learning of the auditory discrimination task. As outlined above, the deactivation of the VP-LHb pathway is expected to decrease LHb output and its excitatory influence on LHb target regions. This finding is consistent with a recent report showing that the optogenetic activation of LHb projections to the medial Raphé nucleus, one of its major efferent targets, triggers a disengagement from ongoing operant behavior.^53^ We suggest that the disengaged state described by Ahmadlou and colleagues may indeed correspond to a DMN brain state. Along similar lines, the inhibition of VP GABAergic neurons or GABAergic VP to LHb projections also triggers task disengagement, such that rats reduce operant behaviors in aversive as well as rewarding contexts.^49^ Finally, lesioning the VP glutamatergic projection to LHb increases task engagement,^54^ weakening DMN activation in similarity to our study.

Cholinergic projections from the VP target the mPFC and LHb, as well as other structures, including the basolateral amygdala.^22^^,^^23^^,^^55^ We observed that the inhibition of cholinergic VP projections improved auditory discrimination performance in both early and late phases of task acquisition. Early and late cholinergic effects thus co-occurred with the LHb and MD pathway-mediated influence of the VP, respectively. Our results are suggestive of coordination between cholinergic and GABAergic VP to LHb projections in the early phase, since these effects occur at similar times and nicotinic cholinergic stimulation has already been shown to potentiate GABAergic neurotransmission in the LHb,^56^ providing a mechanism for the direct interaction of cholinergic and GABAergic pathways. Blockade of LHb muscarinic receptor mediated neurotransmission also promotes goal-directed behavior in a cocaine seeking paradigm,^57^ consistent with the increased task engagement we observed during the optogenetic inhibition of cholinergic VP neurons. In contrast to the LHb pathway, there are no robust direct cholinergic projections from VP to MD, such that the late behavioral effects are likely to be mediated by additional brain areas that receive cholinergic VP input and interact with the MD, such as mPFC or amygdala.^55^^,^^58^ Indeed, none of the three investigated pathways recapitulated the robust decrease in operant lever-pressing we previously observed during the hSynapsin-Arch inhibition of VP cell bodies,^21^ suggesting that this effect is mediated by an additional important influence of the VP not investigated here.

Overall, the inhibition of the VP projection on LHb conferred a learning advantage only during early learning of an attentional task. Once associations between the different auditory stimuli and the reward conditions were established (i.e., during the middle and the late stages), performance was no longer affected by the inhibition of the VP efferents on LHb. While the LHb is not typically seen as a DMN regulatory node, the available literature discussed above is indeed compatible with this notion^35^^,^^53^ and supports the idea that the LHb participates in DMN regulation as per the circuits recruited through VP projections. Regarding the VP to MD pathway, our results are highly consistent with previous findings implicating the MD in DMN regulation,^8^^,^^10^^,^^13^^,^^59^ and confirm these correlative findings using a causal optogenetic manipulation. While at this stage of learning LHb mediated circuits no longer benefit behavioral performance, silencing the MD projection conferred a benefit, possibly by reducing the propensity of reverting to a DMN brain state. Taken together, our data support the notion that subcortical efferent pathways of the VP via LHb and MD contribute to DMN regulation, highlighting the importance of subcortical structures in triggering and maintaining a DMN brain state in coordination with cholinergic neuromodulation.

### Limitations of the study

Some methodological and interpretive limitations should be considered when evaluating these findings. While we successfully recorded from key nodes, including VP, LHb, MD, and medial prefrontal cortex, simultaneous monitoring of all relevant DMN regions was not feasible. This constraint limited our ability to fully map the propagation of VP-driven oscillatory activity across the entire default mode network. Our use of rodent models introduces questions about translational relevance, as DMN organization and function may differ between rats and primates. While the core principles of VP-mediated network modulation are likely conserved, the specific pathway contributions and behavioral manifestations observed here may not directly translate to human cognition and neuropsychiatric conditions. These limitations underscore the need for future studies employing complementary approaches, including primate models and human neuroimaging, to validate and extend our findings.

## Resource availability

### Lead contact

Requests for further information and resources should be directed to and will be fulfilled by the lead contact, Gregor Rainer (gregor.rainer@unifr.ch).

### Materials availability

This study did not generate new unique reagents.

### Data and code availability

• Data reported in this article are accessible via Dryad (https://doi.org/10.5061/dryad.z612jm6r0), as listed in the key resources table.
• Custom made analysis scripts are used to generate the results and figures are available from the lead contact upon request.
• Any additional information is available from the lead contact upon request.

## Acknowledgments

This work was supported by the 10.13039/501100001711Swiss National Science Foundation (grants no. SNF182504 and SNF212255 to G.R.). We thank Dr. Pilar Sanchez for assistance with brain slicing, Nissl staining, and epifluorescence imaging.

## Author contributions

E.-A.M., M.-E.K., M.H., and G.R. designed the experiments. E.-A.M., M.-E.K., M.H., and G.R. analyzed the data. E.-A.M. and M.-E.K. conducted the experiments. E.-A.M., M.-E.K., M.H., and G.R. wrote the article.

## Declaration of interests

The authors declare no competing interests.

## STAR★Methods

### Key resources table

**Table:** 

REAGENT or RESOURCE	SOURCE	IDENTIFIER
**Bacterial and virus strains**		

ssAAV-5/2-hSyn1-chl-eArch3.0_EYFP-WPRE-bGHp(A)	University of Zurich Viral Vector Facility	N/A
scAAV-retro/2-hSyn1-chI-eArchT3.0-SV40p(A)	University of Zurich Viral Vector Facility	N/A
ssAAV-2/2-hEF1α-dlox-eArch3.0_EYFP(rev)-dlox-WPRE-bGHp(A)	University of Zurich Viral Vector Facility	N/A
ssAAV-5/2-hSyn1-hChR2(H134R)_mCherry-WPRE-hGHp(A)	University of Zurich Viral Vector Facility	N/A

**Deposited data**		

Raw Data	This publication	https://doi.org/10.5061/dryad.z612jm6r0

**Experimental Models: Organisms/strains**		

LE-Tg(Chat-Cre)5.1Deis	RRRC	N/A
Wild Type Long-Evans rats	Janvier Labs	N/A

**Software and Algorithms**		

MATLAB	Mathworks, Natick, MA	N/A

### Experimental model and study participants

#### Animals

The local ethical committee on animal experimentation (canton of Fribourg), approved all experimental procedures. A total of 72 adult Long Evans rats of either sex were used in this study. Rats were maintained on a 12/12 light/dark cycle which was reversed so that rats were trained during their active period. During behavioral training rats were lightly food restricted and maintained within 90–95% of their free feeding weight. Water was available *ad libitum*.

### Method details

#### Viral injections

Anesthesia was induced with ketamine (100 mg/kg) and xylazine (20 mg/kg), and maintained using 1–2% isoflurane in pure O2 inhalation. Animals were fixed in a stereotaxic device, Kopf instruments Tujunga, CA, a midline incision was made on the scalp, and burr holes were made over the injection target(s): VP: AP: −0.84; ML: ±3.1, DV: −7.42, MD: AP: −3.48 ML: ±0.5, DV:-5.9. We used three viral constructs, for the transfection of VP axon terminals in LHb; ssAAV-5/2-hSyn1-chl-eArch3.0_EYFP-WPRE-bGHp(A); for retrograde transfection of MD projecting cell bodies in VP, scAAV-retro/2-hSyn1-chI-eArchT3.0-SV40p(A); for the transfection of cholinergic neurons, ssAAV-2/2-hEF1α-dlox-eArch3.0_EYFP(rev)-dlox-WPRE-bGHp(A), and for anesthetized experiments, ssAAV-5/2-hSyn1-hChR2(H134R)_mCherry-WPRE-hGHp(A). All viruses were obtained from the University of Zurich Viral Vector Facility. In all cases 500 μL of virus was injected at a rate of 100 nL/min using a Hamilton syringe equipped with a 34 gauge needle (WPI, Sarasota, FL) and driven by a syringe pump (Micro4, WPI, Sarasota, FL). Following the injection the needle was left in place for 10 min and then removed. Incisions were closed with suture, and rats were allowed to recover for at least one week prior to behavioral training.

#### Behavioral apparatus

Behavioral sessions were conducted in a custom operant chamber (32 × 32 × 55 cm) equipped with one lever, a house light and a reward light at the feeding trough. The operating chamber itself was housed in a custom sound attenuated chamber. Pellet rewards, 45 mg chocolate flavored sucrose pellets, TestDiet Richmond IN, were dispensed from a pellet dispenser (med Associates, USA). The chamber was equipped with a camera and a speaker mounted 60 cm above the arena. The operant chamber (lever press readout, house light, food light and the reward dispenser) were controlled by a PC with custom MATLAB scripts.

#### Behavioral training-automatized lever pressing

Lightly food deprived, 90–95% body weight, animals were first shaped to press a lever for food reinforcement delivered on a continuous reinforcement schedule (CRF), i.e., the animal is rewarded every time it presses the lever. Training then took place in stages moving from the CRF, through fixed ratio (FR), the animal must press a fixed number of times in order to receive a reward, and finally to a Variable Interval (VI) schedule, where reinforcement is delivered for the first response following a randomized time interval. Once stable, moderate response rates were obtained on a VI30s +/− 5s schedule, animals were kept on this schedule until they had received a minimum of 600 rewards, thus ensuring the transition from goal directed to stimulus/response i.e., automatized behavior.

#### Device implanation

Anesthesia was induced and maintained as described above. A midline incision was made and burr holes were drilled over the target sites as described for the viral injections. A bilateral, wireless, battery free device (NeuroLux, Chicago IL) equipped with side emitting yellow LEDs was then lowered to the desired target locations. The device was fixed in place using a combination of cyanoacrylate (Loctite, Westlake OH) and dental cement (Paladur). The incision was then closed over the device, and the animals were left to recover for at least one week prior to the resumption of behavioral training.

#### Behavioral training - Lever press rate

Following device implantation animals were returned to the VI30s schedule for additional training to ensure responding was still stable. Then, in order to assess the effect of optogenetic inhibition on lever press rate on the VI30s schedule of reinforcement, half of the daily trials, 30/60, were paired with optogenetic inhibition of the relevant area while the other half were left as a control. Trial order was randomized with the caveat that there could be no more than 3 of the same trial type presented in a row, and trial times were equalized between optogenetic inhibition and control trials.

#### Behavioral training - discrimination task

After lever pressing had become automatized on the VI30s schedule, rats were transitioned to an auditory discrimination paradigm, with four types of trials each associated with a particular musical piece (S + _A_, Chopin, Piano; S-_A_, Bach, Cello; S + _B_, Beethoven, March; and S-_B,_ Smetana, Moldau). Each musical piece was equalized for overall spectral amplitude and were played in 8s long loops repeated continuously during each trial. Animals completed daily sessions consisting of 60 trials, whose duration was randomized (60 ± 3 s) and were interspersed with randomized (1 ± 3s) intertrial intervals during which the house lights and music were turned off and rewards were not available. Trials were presented in random order with the caveat that no particular trial type could repeat more than three times in a row, and the duration of each trial type was equalized such that the animals spend an equal amount of time in the 4 conditions. Within rewarded trials (S + _A_ and S + _B_) rats received sucrose pellets for lever presses on the same VI30s schedule used for operant training, whereas on unrewarded trials (S-_A_ and S-_B_) no reward was available. To assess the impact of optogenetic manipulations, light inhibition was delivered during one set of music stimuli, S + _A_/S-_A_ and the other set of stimuli, S + _B_/S-_B_ was left as a control. Discrimination performance was measured as percent correct calculated independently for the light inhibition and control conditions, S+ _A_/(S+ _A_ + S-_A_), and S+ _B_/(S+ _B_ + S-_B_).

#### Electrophysiology anesthetized animals

Anesthesia was induced and maintained as described above, and a midline incision was made over the skull and the periosteum retracted. Burr holes were drilled over the relevant target areas, LHb (AP: −3.6, ML: −1, DV: 4.74), MD (AP: −3.48, ML: −0.5, DV: 5.4), mPFC (AP: 3.7, ML: −0.5,DV: −5), VP (AP: −0.84,ML: −3.1,DV: 6), and the neck muscle served as reference. We used two different recording strategies: one during optogenetic stimulation of VP cell bodies, Figures 1, 2, and 3, and another during the optogenetic stimulation of VP axon terminals in LHb and MD, Figure 4. In both cases data were digitized through a unity gain head stage (Cereplex M, Blackrock, Salt Lake City, UT) and routed through an optical converter to a signal processor, (Digital Hub and Cerebus respectively, Blackrock, Salt Lake City, UT), and stored on a PC for offline analysis. In the first case, *N* = 4 animals, a custom made optrode consisting of a single tungsten electrode (FHC, Bowdoin ME) with a tip resistance of ∼250 kΩ was fixed to a 100 μm diameter optic fiber and lowered to 1 mm above the VP. Then, blue 473 nM pulsatile (5–40 Hz) laser stimulation (Optoengine, Midvale, UT) began and the optrode was slowly advanced until light activated cells in the VP were encountered. Using 3 mW laser power and at a numerical aperture 0.22, irradiance drops to 9.7 mW/mm^2^ at a distance of 0.35 mm from the fiber tip, as 20-70 mW/mm^2^ is recommended for robust optogenetic modulation of neural elements.^60^ Unit recordings were made in LHb using a similar tungsten electrode described above and advanced using a motorized micro drive (NAN Instruments, Nof Hagalil, Israel). In the case of VP axon terminal stimulation in LHb and MD, *N* = 2 animals, one laminar probe 16 channels, 100 mM site spacing, (s-Probe, Plexon, Dallas, TX) was inserted into mPFC, and another s-Probe identical to the first except with a larger diameter and equipped with 3 blue LEDs between recording sites 4/5, 10/11, and 14/15 was inserted into LHb/MD. Thus, we could activate VP terminals along the recording trajectory, see Figures 4C and4D, while simultaneously monitoring unit activity in MD, LHb and mPFC.

#### Behavioral electrophysiology

Rats received initial anesthesia with ketamine (100 mg/kg) and xylazine (20 mg/kg), and anesthesia was maintained using 1–2% isoflurane in pure O2 inhalation. Animals were fixed in a stereotaxic device, and a midline incision was made on the scalp. Burr holes were made over the implantation target(s): PrL: AP: +2.76 ML:+0.5 DV:-4, VP: AP:-0.12 ML: +2.5, DV: −8.2, MD: AP: −3.24 ML: +0.5, DV:-6, AC: AP:-4.68 ML:+6 DV: −4.5 (with a 30 medio-lateral angle). Tungsten microelectrodes (FHC Inc. Bowdoin ME, 400 kU) were inserted in the targets and secured to the skull surface with four stainless steel screws and dental cement. A screw implanted over the cerebellum served as the reference electrode. All microelectrodes and the reference screw were wired to a PCB outfitted with a soldered Omnetics connector and fixed to the animal’s skull using dental cement. All behavioral tests were conducted at least one week following electrode implantation.

Continuous local field potential (LFP) recordings were acquired from four brain regions: PrL, VP, MD and AC using a miniature wireless head stage (MultiChannel Systems ME2100, Reutlingen Germany) at a sampling rate of 25 kHz. Data were collected during both home cage spontaneous activity and experimental control conditions. LFPs were down-sampled to 200 Hz and band-pass filtered for gamma-range analysis. For spectral analysis, the down-sampled signals were segmented into 1-s epochs. Epochs containing artifacts were excluded. Power spectra were then computed for all artifact-free epochs using the Fast Fourier Transform (FFT). All analyses were performed using custom MATLAB scripts (MathWorks, Natick, MA, USA).

#### Data analysis

All data analyses were performed using custom scripts written in MATLAB (Mathworks, Natick, MA).

##### Spike phase locking

Spike phases in relation to the stimulus frequency were calculated as 2*πfspt*, where *f* is the stimulation frequency and *spt* is the spike time in seconds. The null hypothesis was that spike phases were distributed equally around the circle, and this was tested using the Rayleigh statistic, specifically the circ-rtest function in MATLAB circular statistics toolbox.

##### Spike triggered average and spike field coherence

In order to calculate the spike triggered average, STA, we averaged 200 ms epochs of LHb LFPs centered on the VP spikes. For the spike field coherence, SFC, we took the FFT of the STA, and then divided it by the average spectral power calculated from the FFTs of all the epochs used in calculated the STA, $\bar{P}$. In this way the SFC is normalized to the existing power in the cortical LFP. $SFC\left(f\right)=\left[\frac{\text{STA}fft\left(f\right)}{\bar{P}\left(f\right)}\right]$, where *f* is an individual frequency band.

### Quantification and statistical analysis

#### Statistics

For comparing firing rates during opotogenetic stimulation vs. baseline, we applied both Lilliefors and Anderson-Darling tests to the difference in rates between optogenetic and baseline conditions. If both tests failed to reject the null hypothesis that the data were normally distributed, we proceded with paired t-tests. For repeated measures ANOVAs Lilliefors tests were performed on the conditional residuals, which failed to reject normality in all cases.
