# Supplementary material for: Ventral pallidum efferent pathways via mediodorsal thalamus and lateral habenula mediate default mode network regulation
Source: iScience. 2025 Oct 28;28(11):113885. doi: 10.1016/j.isci.2025.113885 (PMC12661436; doi:10.1016/j.isci.2025.113885)
Supplement: Document S1. Figure S1 [file mmc1.pdf]

iScience, Volume *28*

Supplemental information

Ventral pallidum efferent pathways via mediodorsal thalamus and lateral habenula mediate default mode network regulation

Epistimi-Anna Makedona, Mu-En Kuo, Michael Harvey, and Gregor Rainer

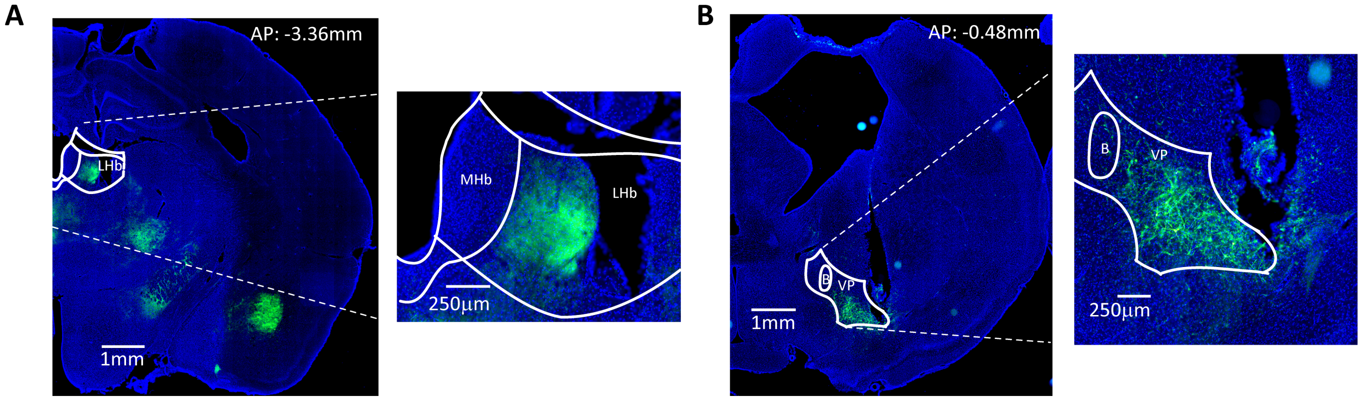

Suppl. Figure 1: *Viral expression near Neurolux device implantation sites.*
(A) Left: Coronal section from the left hemisphere of a WT rat showing Arch/EYFP expression in the VP terminals innervating the LHb, alongside tissue damage caused by the implanted Neurolux device. Note the neurolux device emits light from the side. Right: Zoomed-in view of the same area. (B) Similar images from a ChAT Cre rat injected in the ventral pallidum showing CRE dependent Arch/EYFP expression in the cholinergic neurons of the VP. Right: Zoomed-in view of the same area. B, basal nucleus of Meynert; VP, ventral pallidum; MHb, medial habenula; LHb, lateral habenula
